# Vegetation- and Environmental Changes on Non-Reclaimed Spoil Heaps in Southern Poland

**DOI:** 10.3390/biology9070164

**Published:** 2020-07-15

**Authors:** Oimahmad Rahmonov, Robert Krzysztofik, Dorota Środek, Justyna Smolarek-Lach

**Affiliations:** 1 Institute of Earth Sciences, Faculty of Natural Sciences, University of Silesia, 41-200 Sosnowiec, Poland; dorota.srodek@us.edu.pl (D.Ś.); just.smolarek@gmail.com (J.S.-L.); 2 Institute of Social and Economic Geography and Spatial Management, Faculty of Natural Sciences, University of Silesia, 41-200 Sosnowiec, Poland; robert.krzysztofik@us.edu.pl

**Keywords:** vegetation succession, coal post-industrial sites, soil–vegetation link, anthropogenic soil, urban forest

## Abstract

The study focused on the changes in vegetation and soils on an undeveloped area of coal mine spoil heaps. The process of vegetation changes was evaluated on the basis of historical cartographic materials and fieldwork. Changes of vegetation in nearly 200 years are presented herein. The main purpose of this study is to present an analysis of spatio-temporal changes in vegetation and their influence on soil features. The diversity of ecological species in terms of habitat requirements, tendency of hornbeam communities formation, and the relationship between forest communities and soil features was found. The basic soil properties were examined under selected plant communities (pH, C_org_, N_t_), available forms of elements (P, K, Mg), and as plant nutrients and heavy metal occurrence (Fe, Zn, Mn, Co, Cd, Pb, Sr, Cr, Cu). The soil organic carbon (C_org_) content varied from 3.17 ± 0.007% to 17.7 ± 0.21% and significant differences were observed between sites. The highest total nitrogen (N_t_) content was recorded in the soils of the sites that were represented by *Populus-Betula* stands (0.60 ± 0.01%). Soil acidity (pH) varied greatly, ranging from acidic (pH = 4.1) to weakly acidic (pH = 5.9). The highest value for Mg_avail_ (205.43 ± 0.5 mg·kg^−1^) was noted in the soils under *Calamagrostis epegijos* (L.) Roth community and for P_avail_ (184.07 ± 3.77) and K_avail_ (346.19 ± 2.92 mg·kg^−1^) under the *Quercus-Pinus* stand. On all sites, Zn was a dominant element and its concentration ranged from 526.1 to 1060.4 mg·kg^−1^. Obtained results show how important it is to study the issue of vegetation changes and the formation of the landscape within an industrial city. The described results are important for the management of urban greening issues. Human influence on the disintegration and development of the natural environment is clearly visible. Due to the diversity of former mining areas and their time of creation, the studied area is one of the most important experimental areas for the determination link between vegetation and soil.

## 1. Introduction

Large-scale industrial development leads to significant changes in urban areas, especially in areas rich in natural resources. In southern Poland (the Dąbrowa Basin and Upper Silesia), the period of biggest expansion in industrial area was during the second half of the nineteenth and twentieth centuries [1,2]. Since the second half of the twentieth century, former mining areas and facilities have been developed and used for many purposes, which cause landscape change [3]. Such landscapes are one of the most interesting examples, of “patch-centered mosaics”, in which forested areas also play an important role [4].

Underground and opencast mining, which results in the complete disintegration of biocenoses and soil cover, is among the most drastic human impacts on the natural environment. It concerns the removal of vegetation and the destruction of terrestrial ecosystem and their components such as water network, soil cover, and changes in relief of terrain. Natural resource mining has one of the strongest impacts among all industries, distorting the natural environment globally [5,6] and often causing it to lose its ecological functions [7] as habitat loss and defragmentation. This history of mining has left a lasting legacy on the landscape in the form of mine buildings, coal dumps, tailings, mine shafts, sedimentation tanks, and other anthropogenic surface relief, which can be seen even in the centers of the cities in the many regions.

Studies of the impacts of mining on various measures of environmental quality and reclamation, for cases with coal mine drainage, open-pit, and underground iron mining, have been published for different ecosystems around the world [5,8,9,10]. Furthermore, there is a larger amount of ecological knowledge that can be synthesized to scope mining-related issues related to terrestrial impacts of mining that have previously received little attention. The ecological footprint of mining in a broad sense includes issues such as dioxide carbon emissions, water use, and biodiversity, which can have important regional and global impacts and have well developed procedures for scoping issues [11].

The primary traces is the area directly influenced by the mine(s), processing/rock crushing facilities, tailings areas, buildings, roads, parking lots, and energy transmission network built to accommodate the mines and workers. Some of these impacts are well known, for example, mined soils have higher bulk density and lower porosity, and therefore more erosion and road networks built for mines are a major contribution to changes in ecological and geochemical processes [5,12]. Although most of the studies done to date have concentrated on aquatic impacts, there is a smaller but still significant body of knowledge of mining impacts on terrestrial ecosystems. In such heavily changed and often contaminated medium/habitats, spontaneous vegetation succession and soil forming processes are taking place.

On degraded, former industrial areas that remain untouched for many years without any development or remediation, a process of successive vegetation occurs [13]. This process of vegetation succession in former industrial wastelands has become a subject of research by environmental scientists around the world [14,15,16,17,18,19,20,21,22,23,24]. Many scientists have discussed the use of different types of reclamation strategies on tailings heaps (brownfield sites) and dumps [25,26,27,28,29,30,31]. In non-reclaimed or undeveloped areas, spontaneous successive vegetation has taken place, despite having hostile habitats such as no seed bank, poor amounts of nutrients or differentiation of granulometric compositions [32,33], and also high content of toxic metals in the ground [34,35]. Regardless, these areas are effectively colonized by plants with different environmental demands. Depending on the bedrock characteristics and proximity of the potential biochore, forest and non-forest plant species occur spontaneously [16,36,37]. The forms and sizes of the anthropogenic footprint of the former mining areas are determined by the diverse processes of colonization by the vegetation [38].

The post-mining areas (heaps) differ in terms of their origin, garanulometric composition, and nutrient content as well as trace element content. Therefore, the initiation of succession is conditioned by the ecological plasticity of individual species that are characterized by a high occurrence and amount that gives them a specific set of features (among others such as height, high seed production, biomass, and root features) that help them to compete effectively with other species, which reduces their numbers [38,39]. However, competition during the initial stages of succession is not necessarily intensified due to the availability of varying ecological niches in the spoil heap. An important role in initiating succession in such extreme habitats (heaps) is played by pioneer shrub species, which also affect the formation of later stages of succession. These are called ecological engineers in former mining areas, and they develop further phytocenosis and zoocenosis in extreme habitats [20]. At the initial stages, these organisms do not impact significantly on initiation or on changes of the appropriate formation processes of habitats A large amount of biomass is the starting material for the formation of humus soil horizon. Such species have an impact on rate soil formation and their chemical features [25,26]. In such cases, the relationship between soil and vegetation is clearly observed [40,41,42,43]. Additionally, early-succession organisms have extraordinary adaptability in both physiological and morphological characteristics [40,44].

Despite many studies in this field, the issue concerning the impact of mining on the environment still constitutes a research area. This includes typically cognitive models (e.g., former mining ”path-centered mosaics”) as well as development mechanisms. The problem of planning (or lack of it), is nevertheless equally important in the management of this type area. The paper presents the analysis of two important elements whose development depends on each other in this particular case. In the first case, it was noticed that in most cases, the fall of mining led to abandonment of the land, and only parts found new management methods [3,44,45,46,47,48,49]. In non-reclaimed areas, research was undertaken into the development, diversity of vegetation and soil formation, along with its chemical features under the influence of socio-economic changes. Hence, the goals of this paper were to present an analysis of (1) the spatio-temporal changes in vegetation, and (2) the influence of different types of plant community on soil features in the urban former mining areas.

## 2. Materials and Methods

### 2.1. Study Area

**Figure 1:**
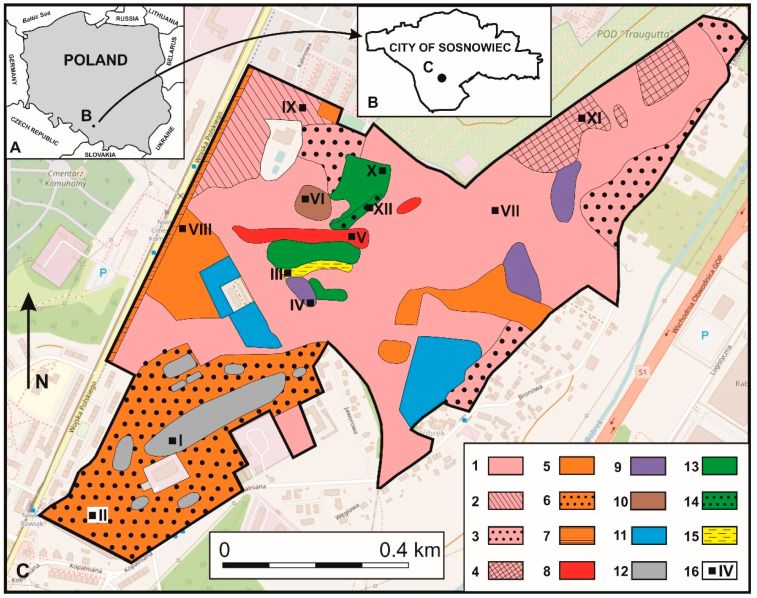
Distribution of vegetation and soil sampling sites: (1) Artificial birch stand (urban park); (2) Betula pendula stand; (3) *Populus-Betula* stands, (4) *Quercus-Pinus* stand; (5) *Robinia pseudoacacia* stand; (6) *Calamagrostietum epigeji*; (7) Thickets of *R. pseudoacacia*; (8) *Juncus effusus* community; (9) Alder community; (10) *Ulmus-Quercus* stands; (11) *Arctio-Artemisietum vulgaris*; (12) *Rudbeckio-Solidaginetium*; (13) *Pinus sylvestris* stand; (14) *Quercus-Pinus* stand; (15) Populus-Alder-Salix stand, (16) Soil sampling places (I–XII). Source: by authors on the base of: OpenStreetMap.org, 2019.

The study area is located in the southern part of Sosnowiec, and covers the location of former mining areas and plants, which were active from around 1800 to 1999 (so called “Lasek”). It is located between 50°15′37.30″ N/19°11′12.50″ E and 50°14′59.90″ N/19°10′07.37″ E as well as between 50°15′01.80″ N/19°09′54.97″ E and 50°15′37.30″ N/19°11′14.62″ E. The most characteristic feature of the studied area are the remains of mining-related infrastructure (such as mine shafts and other mining buildings, mine technical and logistical infrastructure, mine excavations, dumps, and landfill as well as so-called bootleg mining, Figure 1).

The plant site were rebuilt several times from the seventeenth century onwards. This resulted in the formation of an altered land surface layer with a thickness varying from several centimeters to several meters. The primary substrate is comprised of Quaternary glaciofluvial sands and alluvial deposits lying over Triassic carbonate rocks [35].

### 2.2. Analysis of Historical Cartographic Materials

To trace the succession of vegetation in the studied area, the available archival cartographic material, aerial photographs, and surveys from surrounding locations were used. Based on eight maps and one historical aerial photo [50,51,52,53,54,55,56,57,58], the analysis of changes in vegetation and other landscape elements was carried out. Cartographic materials came from the repository of the National Archive in Katowice, and from the personal collections of the authors.

Map analysis and interpretation was carried out using geographical information system (GIS) methodologies, and an interpretative sketch was done in the *MapInfo* geospatial program. The Sosnowiec topographic map from 1958 and the *topographic map of the Upper Silesian Industrial District* from 1960 were georeferenced in two steps. First, the corner points of the raster image were overlaid onto the grid with a size corresponding to the map frame size using affine transformation. Second, rectification was carried out and the image was adjusted to the reference layer using control points. Topographic maps from 1993–1996 and an orthophotomap are available in georeferenced version. The results obtained from the orthophothomaps (from 2015) have been verified and supplemented in the field.

All cartographic materials were digitized and errors usually generated during this operation were detected and eliminated. Screen digitization was combined with the creation of a database of land development. Data included in each series of maps were aggregated to make them comparable [59,60,61]. As a result, vector maps were created that allowed for the comparison of land development in particular time sections to be carried out.

### 2.3. Vegetation Investigation

A survey of the vegetation was conducted from between May and June to the end of September 2019 and mainly involved geobotanical and ecological research. During the research, an inventory of flora was undertaken and phytosociological documentation was also carried out to distinguish the vegetation communities. Furthermore, field studies that involved verification of the actual range of vegetation were conducted. The distinguished plant communities were plotted against a previously prepared sketch. First, the study area was divided into deciduous and coniferous forests and meadows based on the orthophotomap. In order to identify plant communities, phytosociological releves were taken on individual distinguished surfaces using the Braun–Blanquet [62] method. According to this method, to identify vegetation at the association level, the presence of a statement of characteristic and distinctive species for a given association is sufficient.

The average age of vegetation stands was determined based on the age of a dozen trees from the dominant species using a Pressler drill (increment borer). Additionally, data indicating the time of planting of the trees were used, which came from archival records concerning the spatial development of the city [63] and were helpful in distinguishing the development of vegetation stages. During plant mapping, a tape measure, pole, and electronic rangefinder were used for the determination of the range of vegetation patches.

During mapping of the vegetation communities in terrain, a list of vascular plant species were made for each distinguished vegetation community in different stages of succession. Plant species were determined by a key to vascular plant identification [64]. Plant communities were identified based on characteristic species for syntaxonomic units [65,66], or on the more abundant species. Plant names are given according to *Flowering plants and pteridophytes of Poland* [67].

On the basis of various ecological indicator values of vascular plants according to Zarzycki et al. [65], the collected flora were evaluated in terms of ecological parameters such as plant life form, temperature (T), light (L), soil granulometric value (D), resistance to increased heavy metals in the soil (M), and organic content value (H). In this way, the habitat requirements of the analyzed flora were characterized by providing a numerical value (5-degree scale) of given indicators (Supplementary Table S1). On the basis of ecological indicators, the percentage share of flora for selected ecological parameters was calculated (Table S1).

### 2.4. Soil Investigations

Soil samples for chemical analyses were collected only from the humus (A) and organic-humus horizons (OA), which averaged ca. 20 cm in thickness, and represents the main rooting zone of vegetation. The soil was collected in 12 distinguished vegetation communities under the canopy of dominant trees forming plant communities. As the area is subject to constant trampling and is an area heavily penetrated by the inhabitants of the city, for this reason, the soil samples were taken as far as the least transformed surfaces. A total of 36 samples were taken, three under each community. Each sample was analyzed separately and the results for each community were averaged from three samples, and are given in the tables. All samples were analyzed in triplicate for all the properties investigated. Soil analysis did not apply to communities like *Arctio-Artemisietum vulgaris*; *Rudbeckio-Solidaginetium*, and *Populus-Alder-Salix* stands that developed respectively on anthropogenic debris from the mine and wetlands.

Soil samples were submitted for standard physical and chemical analyses: particle size distribution using the sieve analysis method (particle size classes according to Polish Soil Classification 2011); pH in water and in 1 M KCl; hydrolithic acidity (H_h_) according to the Kappen method; organic carbon (C_org_) by the Tyurin method; total nitrogen (N_t_) by the Kjeldahl method; total phosphorus (P_t_) by the Bleck method, modified by Gebhardt [68]; available phosphorus (P_avail_) by Egner–Riehm’s method; and available K_avail_ and Mg_avail_ by PN-R-04023/23 [69].

In the case of the scrub *Robinia pseudoacacia* L., *Ulmus-Quercus* and *Betula-Pinus* stands were examined for the concentrations of selected macroelements (P, Mg, K, Fe, S, Al) and heavy metals (Zn, Mn, Co, Cd, Pb, Sr, Cr, Cu, Ni) in soils were measured using ICP-OES (inductively coupled plasma-optical emission spectrometry) after aqueous mineralization in a solution of nitric and hydrochloric acid (3HCl + HNO_3_). The analyses were made by Bureau Veritas AcmeLabs, Vancouver, Canada.

### 2.5. Statistical Analyses

The Pearson’s correlation rank was applied to check whether there was any relationship between the concentrations of the selected parameters of soils at the investigated sites. Significant differences in the measured soil chemical parameters among the study sites were estimated using the Kruskal–Wallis test. All statistical analyses were done using the Statistica 13.0 package and IBM SPSS Statistics 25. Basic statistic and correlations between soil state indicators were determined at significance levels α = 0.01 and 0.05.

## 3. Results

### 3.1. Vegetation Changes

#### 3.1.1. Changes of Forested Area, 1833–2019

**Figure 2:**
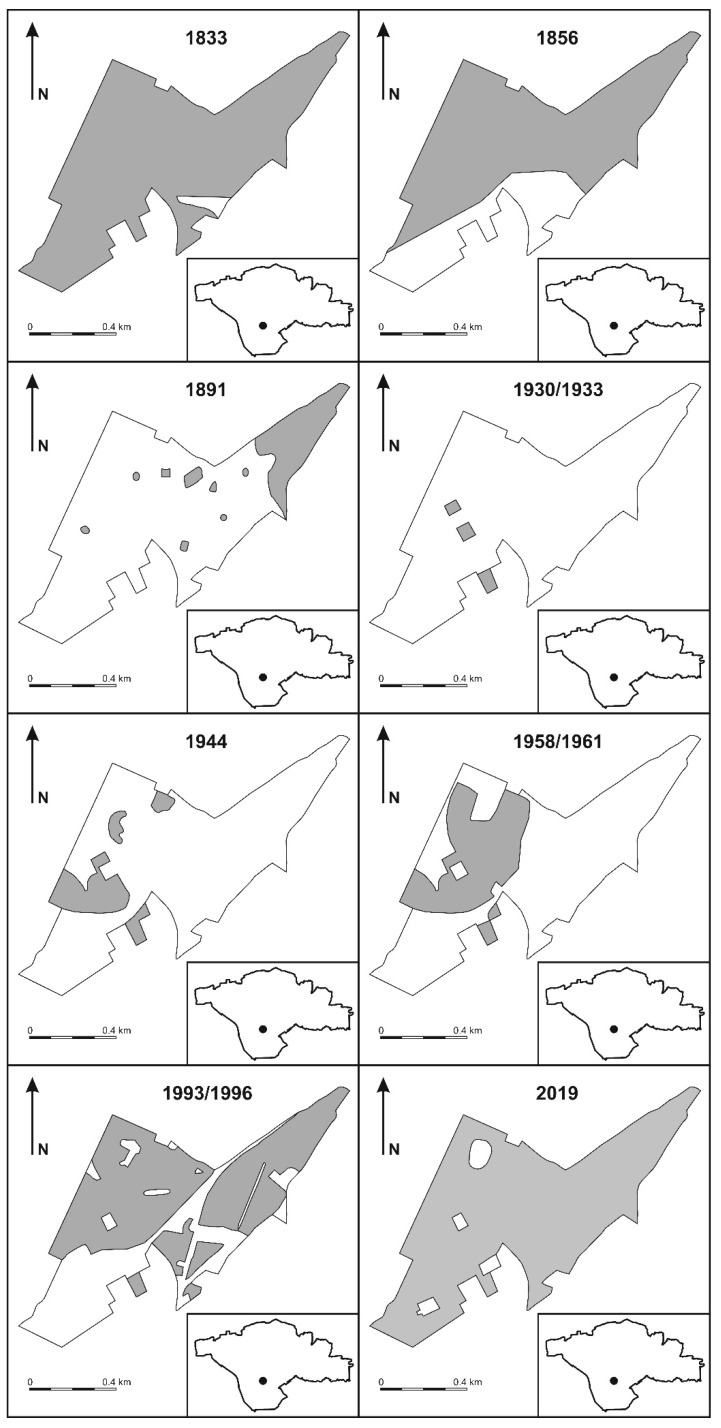
Changes in forested area, 1833–2019. Source: by authors based on historical maps [50,51,52,53,54,55,58] and contemporary field research in 2019.

As can be seen on the map from 1833, in the mid-nineteenth century, the studied area was overgrown by coniferous forests, which are specified in the map legends [50]. In 1856, the period of gradual deforestation had begun due to the expansion of coal mining. By the 1920s, the area was completely stripped of forest. This period of expansion lasted until the beginning of the second half of the 20th century. From that period, the start of reforestation in the area of shallow mining termination can be observed. The last phase of mining activity in the studied area was the closure of the “Niwka–Modrzejów” coal mine in 1999. Demolition of buildings and infrastructure connected with mining led to an expansion in forest succession at the studied area, which occurred as a result of human activity as well as spontaneous succession of vegetation (Figure 2).

#### 3.1.2. Spontaneous Succession

At the studied area, vegetation growth occurred spontaneously on the non-remediated land. Additionally, forest and non-forest vegetation communities developed as artificial plantings resulted (Table 1). Spontaneous ecological succession evolved in open and sunny areas. The leveled former mining surfaces were mostly colonized by *Calamagrostis epigejos* (L.) Roth, *Solidago canadensis* L., and *S. virgaurea* L., which formed plant communities. Arborescent species were represented by *Robinia pseudacacia* L. and *Betula pendula* Roth, which after a certain amount of time formed a stand. At the community border in more loose fragments, *Hypericum perforatum* L., *Symphyotrichum novi-belgii* (L.) G.L. Nesom, *Epilobium angustifolium* L., and other plants could occur (Table 1). On the small surfaces of construction debris, *Rudbeckio-Solidaginetium* and *Calamagrostietum epigeji*, both with an almost complete species composition, developed well (Table 1).

The initial stage begins with pioneering, psammophilous plants such as *Corynephorus canescens* L.P. Beauv., *Heracium pilosella* L., *Herniaria glabra* L., and single seedlings of *Pinus sylvestris* L., which occurred over a small-surface area on a sandy habitat.

Furthermore, the *Juncus effusus* L. em. K. Richt. community formed naturally on the wetland surfaces, which resulted from the occurrence of heavy silt at the surface, which is water-impermeable and allows the formation of such communities. It also includes hygrophilous species such as *Carduus crispus* L., *Phragmites australis* (Cav.) Trin. Ex Steud., *Juncus articulatus* L. em. K. Richt., *Deschampsia caespitosa* (L.) P.Beauv., and at the rim, mesophilic taxons (*Sonchus arvensis* L., *Fragaria vesca* L., *Medicago sativa* L., *M. lupulina* L., *Potentilla anserina* L., *Dactylis glomerata* L., *Vicia cracca* L., *Agrostis canina* L.) grow. Here, species indicating the trend of riparian forest formation with participation of *Alnus glutinosa* (L.) Gaertn., *Populus tremula* L., *Salix rosmarinifolia* L., *S. caprea* L. occurred.

As mentioned earlier, a distinct part of former mining area surfaces is undergoing forest succession, where the initial stage of forestation was formed spontaneously. Pine, elm-oak (*Ulmus laevis* Pall*-Quercus robur* L.), black locust (*R. pseudoacacia* L.) forests and pine coniferous forest appear. The age of the stand is estimated to be 30–70 years. Its spontaneous formation is evident from uneven distribution and population of tree species characterized by different ages. One can often find stubby and clumpy tree specimens where several sprouts grow from one root. Individual forest communities are characterized by different occurrences of tree species (Table 1). Their common feature is the appearance of *Padus serotina* (Ehrh.) Borkh. both in the trees and shrubs layer.

The most diverse in terms of shrub species was the *Ulmus laevis*-*Quercus* assemblage an the initial birch forest where *Euonymus verrucosus* Scop, *Corylus avellana* L., *Prunus spinosa* L., *Prunus cerasus* L., *Padus serotina* (Erkh.) Borkh., *Rhamnus cathartica* L., *Crataegus monogyna* Jacq. and *Viburnum opulus* L. grew. These species are characteristic of dry-ground forests, hence the trend can be considered as a transformation into hornbeam forests.

#### 3.1.3. Secondary Succession

At the studied location, the secondary succession is mainly associated with afforestation by *Pinus sylvestris* L. and *Betula pendula* Roth. Birch afforestation in the western part of the locality during the formation of the city park and recreation area took place during the 50s and 60s of the 20th century. *B. pendula* Roth dominates, whereas another tree species (Table 1) occurred at several places including alien species to Polish flora (*Tilia americana* L., *Quercus rubra* L., *Acer saccharinum* L., *Populus x canadensis ‘Serotina’* Moench, *Pinus nigra* J.F. Arnold, *Robinia pseudoacacia* L.).

The artificial pine forest was very poor in terms of species composition (Table 1) and had a plantation characteristic. The stand was about 60–70 years old. *Padus serotina* (Ehrh.) Borkh. accompanied mainly pine, and *Q. robur* L. and *A. psedoplatanus* L. occurred individually, often reaching the height of the pine.

### 3.2. Flora Diversity

**Figure 3:**
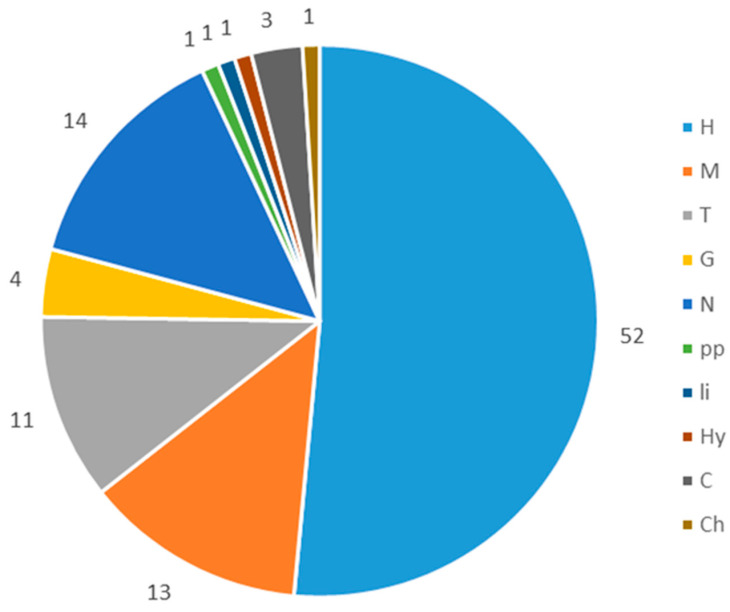
Percentage share of plant life forms in the Niwka postmining area: (H) hemicryptophyte; (M) megaphanerophyte, (N) nanophanerophyte, (T) therophyte; (G) geophytes; (Ch) chamaephytes; (Hy) hydrophytes; (C) herbaceous chamaephyte, (li) liana, and (pp) semiparasite.

As a result of floristic research, 80 vascular plant species were found in the area. The flora of former mining wastelands was dominated by hemicryptophytes (51%), megaphanerophyte (13%), nanophanerophytes (14%), and theophytes (11%). The remaining share of species was 4% (Figure 3).

**Figure 4:**
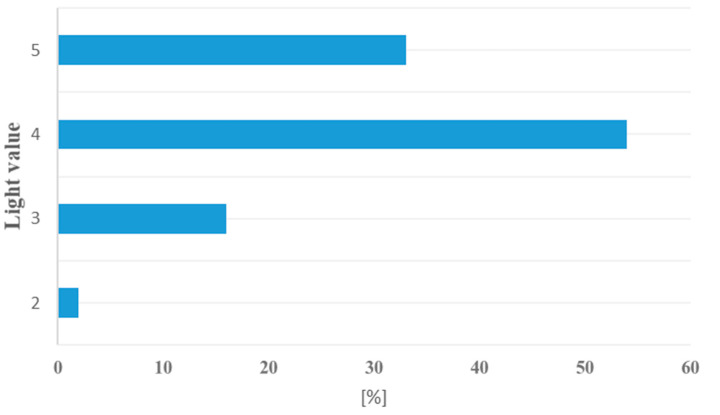
Plant requirement in respect of light values: 2—moderate shade; 3—semi-shade, 4—moderate light and 5—full light.

**Figure 5:**
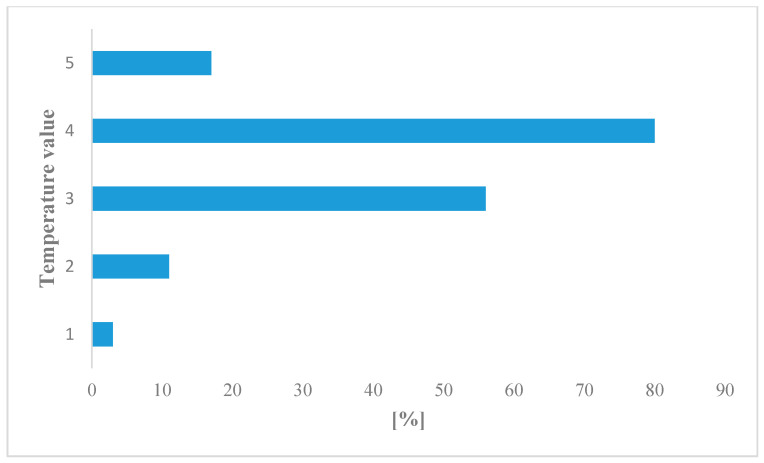
Plant requirement with respect to temperature: (1) coldest climatic conditions, (2) moderately cold areas, (3) moderately cool climatic conditions, (4) moderately warm climatic conditions, (5) warmest regions and microhabitats.

In term of light and thermal requirements, the plant species were not significantly diverse (Figure 4 and Figure 5). Species adapted to moderately warm climatic conditions and constant delivery of moderate light dominated.

In the case of species preferences to soil, granulometric values (D) for the respective species domination was affirmed (Supplementary Table S1) to a habitat type of argillaceous clay and dusty deposits (55%), sandy (25%), heavy clay and loam (15%), and 5% of species adapted in rock debris/scree and gravel. In the event of organic matter content value (H), most species showed preference for a medium with mineral-humic soil (66%), soil rich in organic matter (18%), and other habitats with soil poor in humus (16%, Supplementary Table S1). Such species diversity indicates the mosaic character of the substrate resulting from the diversity of ground grain composition. Soil texture will also determine the soil’s water capacity and nutrient compounds for various groups plant developing in disturbed ecosystems that are mine spoil heap.

In term of resistance to increased heavy metal soil content value (M), 10 species that had increased tolerance of heavy metal content were found: *Calamagrostis epigejos* (L.) Roth, *Cardaminopsis arenosa* (L.) Hayek, *Carex hirta* L., *Cerastium semidecandrum* L., *Erigeron acris* L., *Festuca ovina* L., *Fragaria vesca* L., *Rubus caesius* L., *R. idaeus* L., and *Scabiosa ochroleuca* L.

### 3.3. Chemical Properties of Soils in Sites under Different Vegetation Community

Granulometric composition of the examined soils was diverse (Table 2). In terms of granulometric groups, fine- and medium-grained sands dominated in the samples, and in sample numbers 4, 5, 11, and 12, the silt fraction was significant. The remaining fractions are shown in Table 2.

The C_org_ content of soils varied from 3.17 ± 0.07% (site 6, *Ulmus-Quercus* stand) to 17.73 ± 0.21% (site 12, *Quercus-Pinus* stand), and significant differences according to the Kruskal–Wallis test were observed between sites: 9 to 12 (*p* = 0.008) and 6–12 (*p* = 0.049). The highest N_t_ content was recorded in the soils of the sites represented by *Populus-Betula* stands (0.60 ± 0.01%) and the differences among the sites were significant (*p* = 0.0003, Table 3). The highest content of organic carbon and total nitrogen not only resulted from the decomposition of organic matter, but also from the granulometric composition, especially with the fine sand fraction (0.25–0.1 mm), which had good adsorbing ability.

Soils of the study sites were characterized by a wide range of pH since they can be classified as varying from very acidic through to acidic and weakly acidic (Table 3). The pH values were between 4.1 ± 0.17 and 5.3 in KCl and from 4.6 to 5.9 in H_2_O (Table 3). According to the results of the Kruskal–Wallis test, statistically significant differences (*p* = 0.0012 and *p* = 0.0008, for pH in H_2_O and in KCl, respectively) were recorded between sites 8, 9, and 10. The highest levels of hydrolithic acidity (H_h_) were noted for the soils from site 12 (mean 14.65 ± 0.0.04 c.mol(+)·kg^−1^ (Table 3), and this value was significantly higher (*p* = 0.0004) than those recorded for sites 6 and 7 (4.45 ± 0.04 and 10.67 ± 0.01 cmol(+)·kg^−1^, respectively).

All of the available elements including Mg, P, and K, varied greatly among the investigated sites (*p*-values according to the Kruskal–Wallis test was 0.0003 for each case). The highest value for Mg_avail_ (205.43 ± 0.5) was noted in the soils under the *Calamagrosris epegijos* (L.) Roth community (site 1) and the highest for P_avail_ (184.07 ± 3.77) and K_avail_ (346.19 ± 2.92) was found in the case of site 12 (*Quercus-Pinus* stand).

The content of total phosphorus (Pt) as an indicator of anthropogenically contaminated soils (according Gebhardt), ranged from 142.23 ± 2.35 (site 10) to 593.17 ± 4.56 (site 7) at the analyzed sites (1–12). Significant differences according to the Kruskal–Wallis test were observed between sites: 7–10 (*p* = 0.013), 9–12 (*p* = 0.008) and 6–12 (*p* = 0.049) and significant differences (Table 4) according to the Kruskal–Wallis test were observed between sites: 10–12 (*p* = 0.049), 10–7 (*p* = 0.013) and 6–7 (*p* = 0.021). The occurrence and high content of total phosphorus in the analyzed region were closely related to domestic and industrial waste. Part of the areas by residents are used as a landfill (illegally) and free range for animals, which indirectly affects the content of this element in top soil horizons. The urban shrinkage has meant that many species of larger mammals have found shelter in this small forest and could also indirectly affect habitat fertility.

### 3.4. Correlation Coefficient in Sites

The Pearson’s correlation coefficient showed a significant, positive relationship between the soil parameters (C_org_, N_t_, Mg_avail_, K_avail_, P_avail_, and P_t_) within the analyzed sites. In each case, high correlation between C_org_ and N_t_ in site 8 (*r* = 0.9995 (*p* = 0.021) was confirmed (Table 5). In sites 6 and 7, there were correlations between organic carbon and available potassium (*r* = 0.9972, *p* = 0.048; *r* = 0.9998, *p* = 0.012, respectively), while for total phosphorus at places 1, 8 and 10, the correlation coefficient was *r* = 0.9986 (*p* = 0.034), *r* = 1.0000 (*p* = 0.002), and *r* = 0.9999 (*p* = 0.010), respectively. There was a strong correlation between available magnesium, phosphorus, potassium, nitrogen, and total phosphorus (Table 5). This is primarily due to the flow and decomposition of organic matter and the release of nutrients to the humus horizon.

There was a correlation that was the result of the occurrence of a significant amount of main elements in plant tissues that were released after their mineralization. This is the result of biogeochemical processes rather than soil processes.

### 3.5. The Contents of Selected Macro Elements and Heavy Metals

In terms of contents within macroelements, iron ranked first in all tested samples. The highest amount was found at site 2 (thickets of *R. pseudoacacia* L.) and totaled 19,000 mg·kg^−1^, whereas the lowest content (13,300 mg·kg^−1^) was found in soils under the artificial pine stand community. Total phosphorus values (P) determined by the ICP-OES method ranged from 290 mg·kg^−1^ (site 10) to 580 mg·kg^−1^ (site 9). The amount and quality of the elemental composition in ecosystems depend on the composition of the plant species, and in monocultural ecosystems, depends on the composition of the stand. The rank of macroelement metal concentrations in the humus horizon are given below:

Site 2—Thickets of *R. pseudoacacia*: Fe > Al > Mg > S > K > P
Site 6—*Ulmus-Quercus* stands: Fe > Al > Mg > K > S > P
Site 9—Artificial birch stand: Fe > Al > Mg > K > S > P
Site 10—Artificial pine stand: Fe > Al > Mg > S > K > P

The heavy metal contents in the studied sites were spread amongst the analyzed communities (sites 2, 6, 9, and 10, Table 6). In all samples, the Zn amount was the highest ranging from 526.1 to 1060.4 mg·kg^−1^. The highest concentration of Cu, Mn, Sr (45.88, 509, and 34.4 mg·kg^−1^, respectively) and Ni and Co (22.7 and 11.7 6 mg·kg^−1^, respectively) was under thickets of *R. pseudoacacia* L. (site 2) and *Ulmus-Quercus* stands (site 6) communities. In the artificial birch stand (site 9) and pine stand (site 10) communities, the highest concentrations of Zn (1060.4 mg·kg^−1^), Pb (403.66 mg·kg^−1^), and Cd (11.99 mg·kg^−1^) was noted.

The rank of the decreasing content of heavy metals in the soils from the study sites is as follows (Table 6):

Site 2—Thickets of *R. pseudoacacia* L.: Zn > Mn > Pb > Cu > Sr > Ni > Cr > Co > Cd
Site 6—*Ulmus-Quercus* stands: Zn > Mn > Pb > Cu > Ni > Cr > Sr > Co > Cd
Site 9—Artificial birch stand: Zn > Pb > Mn > Cu > Sr > Cr > Ni > Cd > Co
Site 10—Artificial pine stand: Zn > Pb > Mn > Cu > Sr > Ni > Cr > Cd > Co

## 4. Discussion

Referring to the issues indicated in the introduction regarding forest areas located in post-mining areas, it should be noted that the analyzed area is a good reflection of these issues from the point of view of environmental changes in Poland and countries with similar post-industrial areas.

Biocenotic systems belong to dynamically changing elements of the geosystem, hence each of them sooner or later will be replaced by another. Changes in the ecosystems are caused by various factors, both natural and anthropogenic. The ability to change is one of the most important ecosystem feature, resulting from the open system of energy and matter in circulation [38].

Such systems include, among others, undeveloped wastelands and their process of regeneration depends primarily on the type of bedrock/gangue material and its properties [35,42,70,71]. Waste rock with a significant amount of nutrient elements and a small or medium fraction promotes the colonization of vegetation [72,73,74], which is associated with high water capacity and aeration. The analyzed area of the substrate is characterized by the features mentioned, hence the formation of the forest community takes place relatively quickly in comparison to different dump types areas.

### 4.1. Vegetation Change in Base of Cartographic Analyses

On the examined area map from 1833 [50], it was marked as covered by coniferous species, but without providing species names. The map of Poland’s potential vegetation shows that the area was overgrown with fertile deciduous forests such as *Tilio-Carpinetum*, *Dentario enneaphylli-Fagetum*, *Querco-Pinetum* on hills, and *Fraxino-Alnetum* in wetlands [66]. Research has shown, that in the eastern part of the studied area, the plant communities referring to species composition and habitat characteristics with oak-hornbeam forests (*Tilio-Carpinetum*) formed. It can be assumed that, previously, this area may have been covered by mixed forests. Such communities in similar areas were also noted in the Czech Republic [14,21,23].

The surface of the former coal mine “Niwka–Modrzejów” and areas of exploitation located north of it, are differentiated by topography. The southern part is basically plain, and the central part is uneven and rolling, which is a result of the excavation of the installed utilities on the land. Small but numerous hills and depressions occur in the studied area. These depressions constitute various habitats and favor the encroachments of various species with different ecological requirements. Various topography is also caused by debris appearance and dumps are overgrown with deciduous tree species.

There is no information on the vegetation transformation in the studied area. Cartographic materials show that in the 1920s and 1930s, this location was almost completely deforested (Figure 2), which was related to surface preparation for coal mining by the shaft method and logging for other economic and communal purposes. Based on the cartographic sources, it can be considered that one beginning of the vegetation succession was in the 1940s, and during this period, strong infrastructural urbanization around this area took place: new housing estates, industrial factories, and a large municipal cemetery were created (small amount of vegetation). The studied area was also a leisure zone for the local people in the second half of the 20th century. Several roads and paths led through the forest, where miners and residents went to work, and to service and commercial facilities located in neighboring estates. It also both positively and negatively affected the landscape succession rate. The ecosystem functioning lasted in this form until the early 1990s. Since that time, due to urban shrinkage, human pressure has been lowered in this area [13,75]. Additionally, the mine liquidation and trade development in all neighboring districts caused the disappearance of pedestrian traffic on the paths. Forests were no longer exploration zones for children and young people from neighboring estates, for whom they were an important alternative to spend their free time in the Communist period. Within the forest, the mining railway tracks, which until recently was a barrier to vegetation, have been almost completely removed. Intense succession has begun, and paths, even forest roads and railways, has begun to be overgrown. In many places, bush layers of the forest have thickened. Trees of several meters in height have already grown, even on the sites of demolished mining facilities. The forests have also been used as many recreational places for children and young people, which was intensively exploited in the 1970s and 1980s. In the Communist period (before 1989), this area had a large industrial plant surrounded by forest complexes in the form of patch-centered mosaics [4].

### 4.2. Spontaneous and Secondary Succession

As in other former mining areas, in non-reclaimed areas, the vegetation succession is mainly initiated by *Calamagrostis epigejos* (L.) Roth. This grass is characterized by high ecological plasticity, and a wide spectrum of nutritional requirements. Its extensive root system supports the absorption of nutrients not only from the ground, but also from atmospheric precipitation. The rapid colonization process in coal mine spoil heaps leads to turf formation on the soil surface, which in turn inhibits erosive processes, but also slows down the recruitment of other plant species. *C. epigejos* (L.) Roth is distinguished by a vegetative life strategy type that contributes to its rapid growth in large areas [76,77,78]. In most heaps and post-industrial areas in Poland and Europe, this species initiates habitat-forming processes by creating thick turf while inhibiting biodiversity [33]. Annual biomass production of this species leads to an increase in organic matter amount on a poor substrate surface after plant decomposition. In subsequent years, it is able to independently create an organic level consisting of this species’ detritus, which is important in enriching the soil and consequently, providing nutrients to other species.

Kozińska and Greinert [79] provide general information on the succession in this area. It shows that the southern and central part of the area is covered by ruderal and synanthropic vegetation, with a predominance of silver birch, black locust, white willow, ash-leaved maple, balsam poplar, and common sea-buckthorn in the tree layer. It can be presumed that these are communities of *Calamagrostietum epigeji*, *Rudbeckio-Solidaginetium*, *Robinia pesudacacia* L., and *Betula pendula* Roth scrubs and black locust forests (Table 1). Currently, these communities occur in the eastern part of the described area. The above-mentioned authors also proposed the concept of reclamation of this locality, which in effect were not reclaimed.

In the eastern and central parts of the area, forests with a significant amount of *Betula pendula* Roth and *Robinia pseudoacacia* L. were formed due to spontaneous succession. The current forest consists of old specimens of *R. pseudoacacia* L. Many of the specimens died through wind or other causes (a shallow root system due to the presence of a high fraction gangue). *P. serotina* (Ehrh.) Borkh., *Q. rubra* L., *Tilia cordata* Mill., a few *C. avellana* L. populations, and young specimens of *Carpinus betulus* L. accompany this community. In terms of species composition, this fragment refers to the oak-hornbeam forest. Its species composition is diversified, and results from various natural and anthropogenic conditions. Individual large specimens with a distorted habitat testify to their spontaneous nature. Often, such specimens develop on anthropogenic raised landforms characterized by better water–air relationships. Under such habitat conditions, an initial forest develops with the participation of *Ulmus laevis* Pall. and *Quercus robur* L. In the depressions, the surface rainwater accumulates periodically and stagnates for a long time, which is not conducive to germination and species development.

The initial pine forest that develop spontaneously occupies small areas at the edge of open sites with a *Quercus robur* L. admixture. The significant share of nitrophilous species (*Chelidonium majus* L., *Urtica dioica* L., *Artemisia vulgaris* L., *Rudbeckia laciniata* L., *Solidago canadensis* L.) in the forest and at its edges is closely related to littering.

The area is also crossed by ditches after infrastructure and basins caused by the collapse of the terrain. In the basins, rainwater stagnates temporarily, which affects habitat diversity and plant species. As a result of spontaneous succession, initial *Fraxino-Alnetum*, with almost a full species composition, forms along the ditches and larger basins. This community has a cluster of natural occurrences. In the stand, decaying specimens of *Salix alba* L. and *S. fragilis* L. occur, which may also indicate spontaneous development of this artificial former mining ecosystem on the geomorphologically diverse area. This affects the way of forming different types of habitats and micro-habitats, which determine the rate of succession.

In the former mining areas, multiway reclamation mostly takes place. This applies to both opencast mines and coal dumps [42,43,70]. On non-reclaimed fragments natural succession occurs and the features of rock material storage has a significant impact on its process [16,32,37,39]. Therefore, in each separate area, succession can evolve in different directions taking into account the features of the geochemical gangue. Their common feature is the initiation of succession by ruderal species at the initial succession stages such as *Calamagrostis epigejos* (L.) Roth, *Solidago canadensis* L., and *S. virgaurea* L. in most coal mining areas in Europe [21,34,80].

### 4.3. Differentiation of Soil Properties

The mine area is definitely anthropogenic; soils was transformed significantly by human activity, which caused changes in soil cover, which thereby lost the primary genetic horizons [60]. The soil profile was mixed with post-mining wastes and numerous wastes of other origins. Texture of soils is a result of accidental deposition of materials with the grain composition of sands, clays, and silts with significant (in most places) addition of geogenic coal and coal dust. The soil is classified in accordance with the World Reference Base—WRB [81] as anthropogenic soil of industrial types. The chemical and physical properties such as pH, Hh, Corg, and nutrient compounds (Mg, K, P) for plants (Table 3) are similar to other anthropogenic habitats [70,82]. Variations of soil particle size distribution with land-use types influence soil organic carbon, nitrogen, and other properties of soil [37,64]. In areas that are constantly under human pressure, the water–air relationships of soil are changing. The amount of nitrogen and phosphorus increases by delivering it through diverse human activities.

In pedological research on anthropogenic soils, an extremely important geochemical indicator is total phosphorus content, which is determined by the Bleck method in the Gebhardt [68] modification. Its high amount (above 200 mgPt·kg^−1^) in soil indicates the anthropogenization of the ecosystem. In the studied area, this indicator ranges from 142.23 ± 2.35 to 593.17 ± 4.56 mg·kg^−1^ and at five sites (2, 4, 5, 6, and 7) exceeded the threshold level. Such high values are linked to household and other organic wastes disposed of in this area. High amounts of phosphorus had positive correlation with Corg, Nt, Mg, P, and K available forms (Table 5). Such dependencies were also found by comparing pairs of individual positions and their significant differences at α = 0.05 levels, which were calculated by the Kruskal–Wallis test. The occurrence of these elements is more often associated with plant litter degradation than as a result of soil processes. Mass occurrences of *Padus serotina* (Ehrh.) Borkh. is characterized by high viability and biological production. The organic horizon forming under it is slowly decomposed and the necessary nutrition compounds for plants are released. Hence, in such initial areas, a clear relationship between the above-ground plant biomass (phytomass) and different levels of N, P, Fe, Mg, Al, S, or organic matter is observed [83]. In soils under thickets of *R. pseudoacacia* L., *Ulmus-Quercus* stands, artificial birch stand communities have higher amounts of P, Fe, Mg, and Al than that occurring in artificial pine forest. This happens due to the annual leaf delivery and their fast mineralization.

### 4.4. Heavy Metal Concentration

Among the analyzed heavy metals in all tested samples, Zn (1060.4 mg·kg^−1^) and Pb (403.66 mg·kg^−1^) were dominant. The relatively high heavy metals concentration, which exceeded the admissible value in industrial areas, was only measured for zinc (Table 6). The pollution with Zn in the analyzed areas was higher than in urbanized areas of large European cities like Turino [84], Madrid [85], Warsaw [20] and Sosnowiec [86]. Higher concentrations of zinc are related to the presence of aluminosilicates and spinels containing zinc absorbed in their structure as well as Zn present in materials used for the construction of embankments. Its relatively high values was associated with the presence of a zinc smelter in the adjacent area. The other concentrations of heavy metals were in the permissible range of the standard for farm and urban areas [87].

## 5. Conclusions

Biocenoses undergoes changes temporally and spatially, both under the influence of anthropogenic and natural conditions. The changes in vegetation over nearly 200 years are presented herein. Human influence on the disintegration and development of the natural environment is clearly visible. Due to the diversity of former mining areas and their time of creation, the studied area is one of the most important experimental areas for this type of research. In other places in Upper Silesia and the Dąbrowa Basin, forested former mining areas are more homogeneous and arose at one time (were created for over 200 years on less than 1 km^2^). These changes occurred as a result of spontaneous succession and land reclamation through *Pinus sylvestris* L. and *Betula pendula* Roth planting.

The non-reclaimed area, where succession occurred spontaneously, is characterized by richer species diversity, the diversity of ecological species in terms of habitat requirements and forest formation in the direction of hornbeam tendencies where the relationship between the former forest communities and the nature of the soil is clearly visible.

Soil chemistry showed variations within the individual studied patches. Its diversity is conditioned by the mosaic nature of the habitat, and the degree of transformation of soil compaction under the influence of human activity. Soils developed under the deciduous stands were somewhat more fertile than under coniferous forest. Despite the fact that soils belong to the Technosols type (containing significant amounts of artifacts), they were not contaminated with heavy metals in the analyzed area and their vicinity.

The presented results lead to the conclusion that former mining areas and other degraded areas should be left to spontaneous regeneration through spontaneous succession. This is how the forest in the studied area was renewed. For the most part, no reclamation operations have been carried out, and paradoxically, today, it is even more diverse than the pine forest growing before the era of mining development. Although spontaneous succession is a long-term process, it is very important in the formation of natural biocenotic systems. Such experiences in the renewal of anthropogenically modified ecological systems would be justified and desirable in the evolution of urban ecosystems.

Forest ecosystems often form at urban cores on former mining areas as an important and reviving element of the post-industrial landscape. They also strongly highlight the ‘patch-centered mosaics’ model as the expected model of urban landscape development (townscape). They should be an important attribute during urban planning preparation, which includes sustainable development. In shrinking cities such as Sosnowiec, they can be incorporated into future visions of the city as an element that redefines their spatial and functional structure.

## Figures and Tables

**Table 1:** The main types of vegetation during the succession.

Succession Stage	Type of Vegetation	Habitat Features and Others Remarks
Spontaneous succession	Psammophilic turf—mostly *Corynephorus canescens, Heracium pilosella, Hernaria glabra, Oenothera biennis, Trifolium arvense, Berteroa incana*	Sand pit with a patch of loose sands, open area.
	Community of *Juncus effusus, Carduus acanthoides*, *Phragmites australis*, *Juncus articulatus*, *Deschampsia caespitosa,* on its periphery, *Sonchus arvensis, Fragaria vesca, Medicago sativa, M. lupulina Potentilla anserina, Dactylis glomerata, Vicia cracca, Agrostis canina* occur. Young specimens of trees and shrubs of *Alnus glutinosa, Populus tremula, Salix rosmarinifolia, Salix caprea*	Impervious soils (heavy clays), periodic rainwater collects, wetland habitat.
	*Calamagrostietum epigeji* forms mostly monospecific, compact community, *Hypericum perforatum* and *Symphyotrichum novi-belgii* occur rarely	Dry, sunny terrain on post-mining debris characterized by various fractions and compaction degree (from loose to cemented).
	*Rudbeckio-Solidaginetium: Rudbeckia laciniata, Solidago canadensis*	
	Scrubs of *Robinia pesudacacia* and *Betula pendula (3–4 m tall),* groundcover is covered by *Calamagrostietum epigeji*, sometimes individually *S. virgaurea occurs*	
	*Arctio-Artemisietum vulgaris: Arctium tomentosum, Artemisia vulgaris, Solidago virgaurea, Arctium tomentosum, Verbascum nigrum, Epilobium angustifolium, Urtica dioica, and Chelidonium majus*	Former orchard, open area with construction debris, organic and mineral hills.
	Initial birch forest: Tree layer: *Betula pendula, Quercus robur, Quercus rubra, Robinia pseudoacacia, Populus tremula, Pinus nigra, Ulmus laevis, Acer platanoides, Acer pseudoplatanus, Alnus glutionosa* Shrub layer: *Euonymus europaeus, Coryllus avellana, Prunus spinosa, Prunus cerasus Padus serotina, Rhamnus cathartica, Crataegus monogyna, Viburnum opulus.* Herbaceous plants: *Viola reichenbachiana, Dryopteris filix-mas, Geum urbanum, Vinca minor, Rubus idaeus, Chelidonium majus, Reynoutria japonica*	Anthropogenic field form, where natural succession occur (it was not reclaimed). Birch is co-dominant with other species (Figure 2).
	Initial pine forest: Tree layer: *Pinus sylvestri, Betula pendula, Quercus robur, Quercus rubra, Robinia pseudoacacia, Acer pseudoplatanus.* Herbaceous plants: *Maianthemum bifolium, Geum urbanum, Equisetum sylvaticum*	Flat surface, covered by poorly decaying pine fall.
	Initial elm and oak forest: Tree layer: *Ulmus laevis, Quercus robur, Carpinus betulus, Acer platanoides, Acer pseudoplatanus, Betula obscura, Aesculus hipocastanum* Shrub layer: *Corylus avellana, Prunus serotina, Crataegus monogyna, Cornus sanguinea, Sambucius nigra.* Herbaceous plants: *Viola reichenbachiana, Dryopteris filix-mas, Geum urbanum, Vinca minor, Rubus idaeus, Chelidonium majus*	Slope of the military bunker with 16 m height.
	Alder riparian forest (Fraxino-Alnetum): *Alnus glutinosa, Betula pendula, Salix alba, Salix fragilis, Salix purpurea, Salix caprea, Padus avium.* Groundcover: *Lysimachia vulgaris, Solanum dulcamarum, Potentilla anserina, Lycopus europaeus, Eupatorium cannabinum, Deschampsia caespitosa*	Terrain depressions of various sizes, impermeable substrate.
	Black locust forest: Tree layer: *Robinia pseudoacacia, Quercus robur, Quercus rubra, Tilia cordata.* Shrub layer: *Corylus avellana* and growths of *Carpinus betulus* mostly, individual *Prunus serotina.* Herbaceous plants: *Chelidonium majus, Urtica dioica, Geum urbanum, Convallaria majalis, Viola minor*	It covered high, raised terrain forms of a loose nature.
Artificial planting	Artificial initial birch forest: Tree layer: *Betula pendula*, *Tilia americana, Quercus rubra, Acer saccharinum, Populus x canadensis ‘Serotina’, Pinus nigra, Robinia pseudoacacia, Acer pseudoplatanus* Shrub layer: *Sorbus aucuparia, Malus sylvestris, Symphoricarpos albus, Spiraea salicifolia* Herbaceous plants: *Taraxacum officinale, Trifolium repens, Trifolium pratense, Polygonum aviculare, Melandrium album, Lolium perenne, Achillea millefolium, Solidago virgaurea, Geranium phaeum, Ranunculus acris, Aegopodium podagraria, Arctium lappa, Prunella vulgaris, Plantago lanceolata, Plantago major, Rumex acetosa, Artemisia vulgaris*, etc. Small patches of *Lolio-Plantaginetum* formed along the paths in the park	Arranged city park with leveled surface and unfavorable water-air relations in the soil (caused by trampling).
	Artificial initial pine forest: Tree layer: *Pine sylvestris* mostly, *Quercus robur* individually as an admixture, *Acer psedoplatanus* Shrub layer: *Sorbus aucuparia, Padus serotina* and tree growths Herbaceous plants:—individual *Viola reichenbachiana, Maianthemum bifolium, Rubus caesius, Rubus idaeus*	Flat surface covered with poorly distributed pine fall. The population is evenly distributed with specimens characterized by the same age, no undergrowth, poor ground cover.
	Alley with *Robinia pseudoacacia*: *Acer platanoides, Rosa rugosa, Berberis thunbergii, Ligustrum vulgare*	

**Table 2:** Grain-size distribution of the soil samples.

Number of Sites	Texture (%)								
	Soil Skeleton			Fine Earth					
	[mm]								
	>10.0	10.0–5.0	5.0–2.0	2.0–1.0	1.0–0.5	0.5–0.25	0.25–0.1	0.1–0.05	<0.05
1 *	7.5	15.4	16.9	16.2	7.8	11.1	12.8	7.4	4.9
2	0.5	0.2	0.5	2.9	15.5	33.2	30.5	8.9	7.8
3	2.5	0.0	0.3	2.2	15.0	40.9	33.8	3.4	1.9
4	0.0	0.0	2.0	0.8	11.9	31.2	32.4	10.5	11.2
5	0.0	1.1	1.5	3.2	9.6	25.3	35.8	14.1	9.4
6	0.5	0.5	1.2	4.7	19.7	31.3	31.9	6.2	4.0
7	1.2	1.1	0.1	2.6	11.3	24.9	37.9	10.5	10.4
8	0.9	0.0	0.3	2.6	19.3	33.8	28.9	8.3	5.9
9	3.7	0.0	0.3	3.9	17.4	41.4	25.9	3.9	3.5
10	0.0	0.0	0.9	4.1	19.1	33.9	34.3	5.8	1.9
11	0.0	0.4	0.9	0.1	6.5	26.0	29.1	18.8	18.2
12	0.0	0.0	0.2	4.5	9.8	20.5	34.9	16.9	13.2

* Arabic numeral corresponds to the number of sites and name of the vegetation community as in Table 3.

**Table 3:** Soil properties under different vegetation communities in the post mining landscape.

Number of Sites/Vegetation Community		H_h_	pH		Loss Ignition	C_org_	Nt	C/N	Mg Available	P Available	Pt	K Available
		Cmol (+)kg^−1^	H_2_O	KCl	[%]				[mg·kg^−1^]			
1.	*Calamagrostis epegijos*	7.29 (±0.02)	5.7 (±0.19)	5.0 (±0.14)	29.43 (±0.5) ^a.b^	16.84 (±0.2)	0.35 (±0.01)	48.67 (±1.53) ^b^	205.43 (±0.5) ^a^	4.75 (±0.12) ^a.b^	162.5 (±2.59)	188.1 (±2.42)
2.	Thickets of *R. pseudoacacia*	7.55 (±0.04)	5.8 (±0.16)	5.3 (±0.10)	18.64 (±0.25)	10.48 (±0.21)	0.35 (±0.004)	30 (±1.0)	181.87 (±1.64)	26.37 (±0.68)	354.07 (±2.53)	227.69 (±3.97) ^a^
3.	Alder community	5.22 (±0.02)	5.4 (±0.15)	4.9 (±0.15)	7.68 (±0.20)	4.51 (±0.39)	0.2 (±0.01)	23 (±2.00)	105.8 (±2.88)	7.66 (±0.39)	150.63 (±1.66)	45.46 (±0.91) ^c^
4.	*Populus-Betula* stands	10.52 (±0.04)	5.3 (±0.1)	4.7 (±0.06)	18.84 (±0.25)	13.67 (±0.35)	0.6 (±0.01) ^b.c^	22.67 (±0.58)	147.7 (±1.67)	15.26 (±0.72)	366.03 (±4.54)	163.9 (±2.27)
5.	*Juncus effusus* community	6.52 (±0.01)	5.7 (±0.05)	5.3 (±0.1)	17.07 (±0.15)	11.54 (±0.14)	0.34 (±0.01)	33.33 (±0.58)	221 (±1.32) ^b.c^	26.52 (±0.78)	235.27 (±5.11)	193.48 (±2.23)
6.	*Ulmus-Quercus* stands	4.45 (±0.04) ^c^	5.7 (±0.16)	5.1 (±0.11)	6.22 (±0.2) ^a^	3.17 (±0.07) ^a^	0.14 (±0.004) ^c^	23 (±0)	85.23 (±2.97)	14.57 (±0.37)	143.2 (±2.1) ^c^	133.51 (±0.58)
7.	Birch (Betula) stand	10.67 (±0.01) ^a^	5.5 (±0.1)	5.0 (±0.12)	21.74 (±0.25)	9.37 (±0.16)	0.46 (±0.005) ^a^	20.33 (±0.58) ^a.b^	145.27 (±1.66)	62.74 (±2.43) ^b.c^	593.17 (±4.56) ^b.c^	128.8 (±1.75)
8.	*Robinia pseudoacacia* stand	9.31 (±0.02)	4.6 (±0.11) ^b^	4.1 (±0.17)	10.88 (±0.16)	6.92 (±0.07)	0.27 (±0.003)	26 (±0)	26.6 (±1.4) ^c^	33.26 (±2.74) ^a^	189.27 (±1.9)	134.84 (±2.22)
9.	Artificial birch stand	4.07 (±0.02) ^a.b^	5.9 (±0.1) ^a.b^	5.3 (±0.1)	6.33 (±0.15) ^b^	3.2 (±0.11) ^b^	0.09 (±0.003) ^a.b^	36.67 (±2.31)	59.43 (±0.86)	5.41 (±0.49) ^c^	197.27 (±2.23)	62.87 (±1.7)
10.	Artificial pine stand	6.23 (±1.75)	4.7 (±0.11) ^a^	4.2 (±0.18)	6.51 (±0.1)	3.74 (±0.11)	0.16 (±0.01)	23.33 (±1.15)	23.33 (±1.15)	5.9 (±0.36) ^a.b^	142.23 (±2.35) ^a.b^	35.87 (±2.59) ^a.b^
11.	*Acer-Betula* stands	5.18 (±0.03)	5.6 (±0.12)	5.1 (±0.08)	10.22 (±0.2)	5.44 (±0.19)	0.19 (±0.003)	29.33 (±0.58)	29.33 (±0.58)	150.8 (±2.46)	164.83 (±1.56)	183.66 (±2.21)
12.	*Quercus-Pinus* stand	14.65 (±0.04) ^b.c^	5.2 (±0.15)	4.8 (±0.1)	26.52 (±0.4)	17.73 (±0.21) ^a.b^	0.42 (±0.003)	42.67 (±0.58) ^a^	42.67 (±0.58) ^a^	184.07 (±3.77)	473.07 (±5.2) ^a^	346.19 (±2.92) ^b.c^
*p*-value		0.0004 *	0.0012 *	0.0008 *	0.0003 *	0.0003 *	0.0003 *	0.0004 *	0.0003 *	0.0003 *	0.0003 *	0.0003 *

*: The Kruskal–Wallis test. Difference between the twelve sites was statistically significant at (α = 0.05). a—a. b—b. c—c: The Kruskal-Wallis test for pairwise comparisons between sites. Results are statistically significantly different at α = 0.05 (after Bonferroni adjustment for multiple comparisons).

**Table 4:** Pairwise comparisons between sites (statistically significant difference in the Kruskal–Wallis test: α = 0.05).

Soil Properties		Number of Sites	*p*-Value
H_h_ [cmol (+) kg^−1^]		7–9	0.032
		9–12	0.008
		6–12	0.049
H_2_O [pH]		9–10	0.025
		8–9	0.039
Loss ignition [%]		1–6	0.014
		1–9	0.024
C_org_ [%]		6–12	0.014
		9–12	0.019
N_t_ [%]		7–9	0.032
		4–9	0.008
		4–6	0.032
C/N		7–12	0.033
		1–7	0.008
[mg·kg^−1^]	Mg available	1–10	0.032
		5–10	0.008
		5–8	0.032
	P available	1–8	0.032
		1–7	0.008
		7–9	0.032
	Pt	10–12	0.049
		7–10	0.013
		6–7	0.021
	K available	2–10	0.032
		10–12	0.008
		3–12	0.032

**Table 5:** Correlation coefficient and significance level at selected positions.

Soil Parameters/Site Number	1	2	3	5	6	7	8	9	10	11
C_org_/K_avail._							0.9996 (*p* = 0.019)			
C_org_/N_t_							0.9995 (*p* = 0.021)			
C_org_/P_avail._					0.9972 (*p* = 0.048)	0.9998 (*p* = 0.012)				
C_org_/P_t_	0.9976 (*p* = 0.045)						1.0000 (*p* = 0.002)		0.9999 (*p* = 0.010)	
Mg_avail._/K_avail._										0.9991 (*p* = 0.026)
Mg_avail._/N_t_		0.9996 (*p* = 0.019)		0.9971 (*p* = 0.049)		0.9983 (*p* = 0.037)				1.0000 (*p* = 0.006)
Mg_avail._/P_avail._			1.0000 (*p* = 0.005)				0.9995 (*p* = 0.020)		0.9999 (*p* = 0.010)	
N_t_/K_avail._							0.9981 (*p*=0.040)			0.9987 (*p* = 0.032)
P_t_/K_avail._							0.9995 (*p* = 0.020)	1.0000 (*p* = 0.002)		
P_t_/Mg_avail._	0.9986 (*p* = 0.034)	0.9999 (*p* = 0.010)								
P_t_/N_t_		0.9999 (*p* = 0.009)					0.9995 (*p* 0.019)			
P_t_/P_avail._					0.9997 (*p* = 0.015)					

Statistically significant difference at α = 0.05.

**Table 6:** The total contents of the selected heavy metal and macroselement for plant growth.

Number Sites/Vegetation Community	Cu	Pb	Zn	Ni	Co	Mn	Sr	Cd	Cr	P	Mg	K	Al	Fe	S
	[mg·kg^−1^]														
2. Thickets of *R. pseudoacacia*	45.88	266.01	798.9	21.0	7.8	509	34.4	5.96	15.3	480	1400	900	6000	19,000	13,000
*6. Ulmus-Quercus* stands	23.75	170.83	526.1	22.7	11.7	250	16.0	4.57	16.3	390	1400	800	5900	14,000	400
9. Artificial birch stand	36.79	403.66	1060.4	15.5	4.6	243	24.7	11.99	18.7	580	800	600	11,200	19,000	600
10. Artificial pine stand	35.75	300.67	581.2	12.6	4.5	279	15.1	11.66	12.4	290	600	400	5000	13,300	500

## Supplementary Materials

The following are available online at https://www.mdpi.com/2079-7737/9/7/164/s1, Table S1: Vascular flora of the objects under study and their ecological requirement according Zarzycki et al. [65].
