# Supplementary material for: Vegetation- and Environmental Changes on Non-Reclaimed Spoil Heaps in Southern Poland
Source: Biology (Basel). 2020 Jul 15;9(7):164. doi: 10.3390/biology9070164 (PMC7408371; doi:10.3390/biology9070164)
Supplement: Supplementary file 1 [file biology-09-00164-s001.pdf]

Supplementary Materials: Vegetation- and Environmental Changes on Non-Reclaimed Spoil Heaps in Southern Poland

Table S1. Vascular flora of the objects under study and their ecological requirement according Zarzycki et. al (2002).

Species	Life form	Ecological indicator values				
		L	T	D	H	M
Acer pseudoplatanus L.	M	3	3	2	2	·
Acer platanoides L.	M	4	4	4	2	.
Acer negundo L.	M	4	4-5	2-4	2	·
Acer saccharinum L.	M	n.d	n.d	n.d	n.d	n.d
Achillea millefolium L.	H	4	4-3	4	1-2	·
Agrostis canina L.	H	4	·	4	3	·
Alnus glutinosa (L.) Gaertn.	M	3	4	5	3	·
Arctium lappa L.	H	5	4	4	2	·
Arctium tomentosum Mill.	H	4	4	4	2	·
Artemisia vulgaris L.	H	5	4	4	2	·
Athyrium filix-femina (L.) Roth	H	2	4-2	4	2	·
Berteroa incana (L.) DC.	H, T	5	4	3	1	·
Betula pendula Roth	M	4	4-3	3-4	1-2	·
Calamagrostis epigejos (L.) Roth	G, H	4	4-3	3	1	1
Cardamine pratensis L.	H	4	4-3	4-5	2	·
Cardaminopsis arenosa (L.) Hayek	H	4	4-3	3-2	1-3	1
Carduus crispus L.	H	4	4	4-5	2	·
Carpinus betulus L.	M	2/3	4-5	4-5	2	·
Crataegus monogyna Jacq.	N	3-5	4-5	4-5	2-3	·
Carex hirta L.	G	4	4-3	3-4	2	1
Cerastium semidecandrum L.	H, T	5	4	3	2	1
Chelidonium majus L.	H	3-4	4-3	2-5	2-3	·
Cornus alba L.	N	4	4-3	4	3	·
Corylus avellana L.	N	4	5-3	4	2	·
Corynephorus canescens (L.) P.Beauv.	H	4	4	3	1	·
Crataegus laevigata (Poir.) DC.	N	4-5	4-5	4-5	2-3	·
Crataegus monogyna Jacq.	N	3-5	4-5	4-5	2-3	·
Dactylis glomerata L.	H	4	4-2	4	2	·
Daucus carota L.	H	5	5-3	4	2	·
Deschampsia caespitosa (L.) P.Beauv.	H	3-5	4-1	4	2-3	·
Deschampsia flexuosa (L.) Trin.	H	3-4	4-1	3-4	1-2	·
Epilobium angustifolium L.	H	5-4	4-2	3-5	2	·

Erigeron acris L.	H, T	5	5-3	2-5	2	1
Erigeron annuus (L.) Pers.	H, T	5-4	4-3	2-4	2	·
Erigeron canadensis L.	T, H	5	4-3	3-4	2	·
Euonymus verrucosus Scop.	N	3	5-4	1-4	2	·
Euphrasia rostkoviana Hayne	T, pp	4	4-3	4	2	·
Fagus sylvatica L.	M	3	3	3	2	.
Festuca ovina L.	H	4	4-3	3	2	1
Fragaria vesca L.	H	3-4	4-2	4	2	1
Fraxinus excelsior L	M	3	4-3	4-5	2	.
Galium verum L.	H	5	5-3	4-3	2	·
Herniaria glabra L.	H	4	4-3	3-2	1	·
Hieracium pilosella L.	H	5	5-2	2-4	2	·
Humulus lupulus L.	H, li	3	4	4	2-3	·
Hypericum perforatum L.	H	4	5-3	4	2	·
Juncus articulatus L. em. K. Richt.	H	5	4-3	4-5	3	·
Juncus conglomeratus L. em. Leers	H	4	4-3	4-5	3	·
Medicago falcata L.	H	5	5-4	2-5	2	·
Medicago lupulina L.	H, T	5	4-3	2-4	1-2	·
Medicago sativa L.	H	5	4-3	2-4	2	·
Molinia caerulea (L.) Moench	H	4	4	3-5	2-3	·
Oenothera biennis L.	H	5	4	2-3	2	·
Padus avium Mill.	M	3	4-3	4-5	2	·
Padus serotina (Ehrh.) Borkh.	N, M	3-4	4	3-4	2	·
Phragmites australis (Cav.) Trin. ex Steud.	G, Hy	4-5	4-3	3-5	2-3	·
Pinus nigra J. F. Arnold	M	n.d	n.d	n.d	n.d	n.d
Pinus sylvestris L.	M	4-5	4-3	3-4	1-3	·
Plantago lanceolata L.	H	4	5-3	4	3	·
Plantago major L.	H	5	4-2	3-5	2-3	·
Poa annua L.	H, T	5-3	4-1	3-5	2	·
Poa trivialis L.	H	4	4-3	4-5	2	·
Polygonum aviculare L.	T	5	4-3	2-5	1-2	·
Populus nigra L.	M	4	4	3-5	2	·
Populus tremula L.	M	3	4-3	4-3	2	·
Populus x canadensis Moench	M	n.d	n.d	n.d	n.d	n.d
Potentilla anserina L.	H	5	4-3	4	2	·
Prunus spinosa L.	N	4	5-3	4	2	·
Prunus cerasus L.	N	n.d	n.d	n.d	n.d	n.d
Quercus robur L.	M	4	4-3	4	2	·
Quercus rubra L.	M	4	4	3-4	2	·
Rhamnus cathartica L.	N	3-4	4	2,4	2	.
Robinia pseudoacacia L.	M	4	4-5	2-4	2	·
Rubus caesius L.	N	4-5	4-5	3-4	2	1
Rubus idaeus L.	N	4-5	4-5	3-4	2	1
Rudbeckia laciniata L.	H	5-4	4-3	3-4	2	·

Rumex acetosa L.	H	4	4-3	4	2	·
Salix alba L.	M	4	4	4	1-2	·
Salix caprea L.	M, N	5-3	4-3	3-4	2	·
Salix cinerea L.	N	4	4-3	3-5	3	·
Salix x fragilis L.	M	4	4-3	4	2	·
Salix pentandra L.	M, N	4	4-3	4	3	·
Salix purpurea L.	N	4	4-3	2-4	1	·
Salix rosmarinifolia L.	N, Ch	4	4-3	4	3	·
Sambucus nigra L.	N	(5)4-3	4-3	3-4	2	·
Saponaria officinalis L.	H	4	4	2-4	1-2	·
Scabiosa ochroleuca L.	H	5	4-5	3-4	2	1
Senecio viscosus L.	T	4	4-3	3	·	·
Solidago canadensis L.	G, H	4-5	4	2-4	2	·
Solidago virgaurea L.	H	3-4	4-3	4-3	2	·
Sonchus arvensis L.	G,H	5	4-3	4-3	2	·
Symphyotrichum nov-belgii (L.) G.L. Nesom	H	n.d	n.d	n.d	n.d	n.d
Tanacetum vulgare L.	H	5	4-3	4-2	2	·
Tilia americana L.	M	-	-	-	-	-
Tilia cordata Mill.	M	3	4-3	4	2	·
Trifolium arvense L.	T	5	5-3	3-1	2	·
Trifolium pratense L.	H	4	4-2	4	2	·
Trifolium repens L.	C, H	4	4-2	4	2	·
Ulmus laevis Pall.	M	3	4	4	2	·
Urtica dioica L.	H	2-5	4-2	3-4	2	·
Verbascum nigrum L.	H	4	4-3	2	2	·
Viburnum opulus L.	N	3	4-3	4	2	·
Vicia cracca L.	H	4	4-3	4	2	·

Note. Life form: M – megaphanerophytes, N – nanophanerophytes, Ch – arborous chamephytes, C – herbaceous chamephytes, H – hemicryptophytes, G – geophytes, T – therophytes, Hy – hydrophytes and helophytes, li – lianas, pp – semiparasites.

Ecological indices:

L – light (2 – moderate shade, 3 – semi-shade, 4 – moderate light, 5 – full light);

T – thermal (1 – coldest climatic conditions, 2 – moderately cold biotopes, 3 – moderately cold climatic conditions, 4 – moderately warm climatic conditions, 5 – warmest biotopes);

D – soil grain size index (1 – rocks and crevices, 2 – screes, gravel, 3 – sand, 4 – silts and silty deposits, 5 – loams and clays);

H – index of organic matter content in soils (1 – soils poor in organic matter, 2 – mineral-humus soils, 3 – soils rich in organic matter);

M – index of resistance to increased levels of heavy metals (1 – species resistant to increased levels of heavy metals, 2 – species demanding increased levels of heavy metals); dot – index not determined; n.d. – no data
